# The Arabidopsis homolog of human minor spliceosomal protein U11-48K plays a crucial role in U12 intron splicing and plant development

**DOI:** 10.1093/jxb/erw158

**Published:** 2016-04-17

**Authors:** Tao Xu, Bo Mi Kim, Kyung Jin Kwak, Hyun Ju Jung, Hunseung Kang

**Affiliations:** ^1^College of Life Science, Jiangsu Normal University, Xuzhou 221116, Jiangsu Province, PR China; ^2^Department of Plant Biotechnology, College of Agriculture and Life Sciences, Chonnam National University, 300 Yongbong-dong, Buk-gu, Gwangju 500-757, South Korea

**Keywords:** *Arabidopsis thaliana*, minor spliceosome, plant development, splicing, U12 intron, U11-48K.

## Abstract

The minor U12 introns are removed from precursor mRNAs by the U12 intron-specific minor spliceosome. Among the seven ribonucleoproteins unique to the minor spliceosome, denoted as U11/U12-20K, U11/U12-25K, U11/U12-31K, U11/U12-65K, U11-35K, U11-48K, and U11-59K, the roles of only U11/U12-31K and U11/U12-65K have been demonstrated in U12 intron splicing and plant development. Here, the functional role of the Arabidopsis homolog of human U11-48K in U12 intron splicing and the development of *Arabidopsis thaliana* was examined using transgenic knockdown plants. The *u11-48k* mutants exhibited several defects in growth and development, such as severely arrested primary inflorescence stems, formation of serrated leaves, production of many rosette leaves after bolting, and delayed senescence. The splicing of most U12 introns analyzed was impaired in the *u11-48k* mutants. Comparative analysis of the splicing defects and phenotypes among the *u11/u12-31k*, *u11-48k*, and *u11/12-65k* mutants showed that the severity of abnormal development was closely correlated with the degree of impairment in U12 intron splicing. Taken together, these results provide compelling evidence that the Arabidopsis homolog of human U11-48K protein, as well as U11/U12-31K and U11/U12-65K proteins, is necessary for correct splicing of U12 introns and normal plant growth and development.

## Highlight

The Arabidopsis homolog of human U11-48K protein, a small nuclear ribonucleoprotein, is necessary for correct splicing of minor U12 introns, which is crucial for normal plant growth and development.

## Introduction

In higher eukaryotes, two distinct spliceosomes catalyze the splicing of introns from precursor mRNAs (pre-mRNAs), which is crucial for regulation of gene expression in cells (Burge *et al.*, 1999). The splicing of major U2-type introns is catalyzed by the major spliceosome (Moore *et al.*, 1993; Staley and Guthrie, 1998), while the splicing of minor U12-type introns is catalyzed by the minor spliceosome (Tarn and Steitz, 1997; Patel and Steitz, 2003; Zhu and Brendel, 2003; Will and Lührmann, 2005; Turunen *et al.*, 2013). Although the minor U12 introns constitute <1% of all introns found in eukaryotes (Burge *et al.*, 1998; Levine and Durbin, 2001; Zhu and Brendel, 2003; Sheth *et al.*, 2006; Alioto, 2007), the importance of U12 intron splicing has been demonstrated in diverse cellular processes in many organisms, including embryonic development in *Drosophila* and zebra fish (Otake *et al.*, 2002; König *et al.*, 2007; Markmiller *et al.*, 2014), a developmental disorder known as microcephalic osteodysplastic primordial dwarfism 1 and myelodysplastic syndrome in humans (Jafarifar *et al.*, 2014; Madan *et al.*, 2015), isolated familial growth hormone deficiency in humans (Argente *et al.*, 2014), and nonsense-mediated mRNA decay (Zhu and Brendel, 2003; Hirose *et al.*, 2004).

Assembly of the spliceosome during the splicing process requires the formation of protein–RNA, protein–protein, and RNA–RNA complexes. The major spliceosome consists of U1, U2, U4, U5, and U6 small nuclear ribonucleoproteins (snRNPs), while the minor spliceosome consists of U11, U12, U5, U4atac, and U6atac snRNPs (Hall and Padgett, 1996; Tarn and Steitz, 1996a, *b*; Kolossova and Padgett, 1997; Incorvaia and Padgett, 1998; Will and Lührmann, 2005). More than 200 proteins are found in both the major and minor spliceosome complexes. In addition to the many proteins present in both spliceosomes, seven proteins unique to the minor spliceosome, denoted as U11/U12-20K, U11/U12-25K, U11/U12-31K, U11/U12-65K, U11-35K, U11-48K, and U11-59K, have been identified in animals and plants (Will *et al.*, 2004; Lorković *et al.*, 2005). In plants, these proteins were found to be highly conserved in dicotyledons and monocotyledons (Will *et al.*, 2004; Lorković *et al.*, 2005; Russell *et al.*, 2006). These minor spliceosome proteins are known to associate with the small nuclear RNAs (snRNAs), denoted as U11, U12, and U4atac/U6atac snRNAs (Will *et al.*, 2004), a process which is necessary for recognition of the 5' splice site, 3' splice site, and branch-point site during spliceosome formation (Tarn and Steitz, 1996b; Frilander and Steitz, 1999; Turunen *et al.*, 2013). It was previously demonstrated that human U11/U12-65K protein interacts with U12 snRNA and recognizes branch-point sites (Benecke *et al.*, 2005). A recent study also demonstrated that Arabidopsis (*Arabidopsis thaliana*) U11/U12-65K interacts with U12 snRNA, which is necessary for U12 intron splicing and plant development (Jung and Kang, 2014). Through functional analysis in Arabidopsis and rice (*Oryza sativa*), U11/U12-31K was found to be indispensable for U12 intron splicing, and crucial for the normal development of dicot and monocot plants (Kim *et al.*, 2010; Kwak *et al.*, 2012). All of these studies clearly demonstrate that the proteins unique to the minor spliceosome complex are necessary for U12 intron splicing and development in both animals and plants.

Although the aforementioned studies clearly indicate the importance of minor spliceosome proteins in U12 intron splicing and development of plants, the significance of most minor spliceosome proteins has not yet been proven experimentally. The Arabidopsis homolog of human U11-48K (At3g04160), investigated in the present study, is one of the proteins unique to the minor spliceosome complex. It was previously demonstrated that human U11-48K protein interacts with the U11 snRNA and recognizes the 5' splice site of U12 introns, which is important for U12-type splicing and cell growth (Turunen *et al.*, 2008; Tidow *et al.*, 2009). However, the cellular role of the Arabidopsis homolog of human U11-48K in the splicing of U12 introns, and the consequences of U12 intron splicing in plant growth and development, has not yet been determined. In this study, we provide evidence that the Arabidopsis homolog of human U11-48K is necessary for the correct splicing of U12 introns, which is crucial for normal growth and development of plants.

## Materials and methods

### Plant materials and growth conditions

**Fig. 1.**
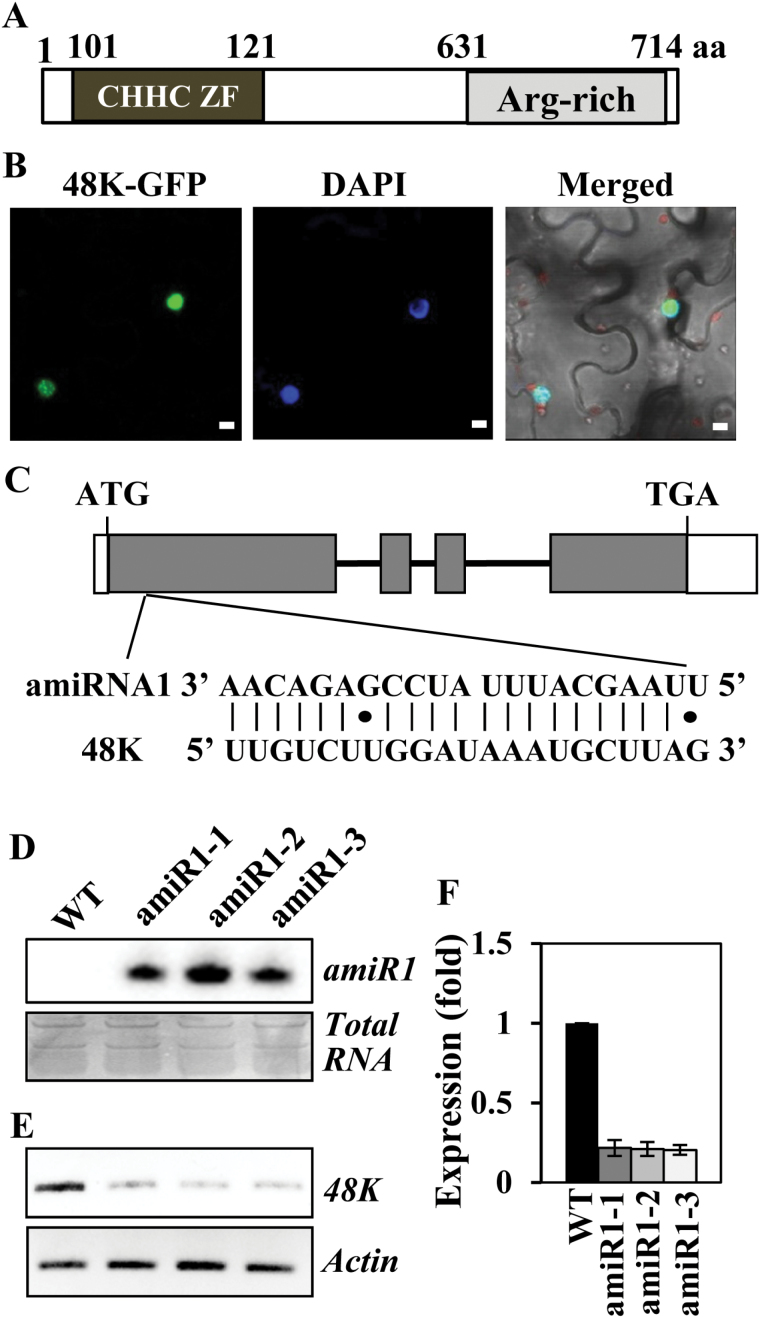
Domain structure and cellular localization of the Arabidopsis homolog of human U11-48K protein and generation of artificial miRNA-mediated knockdown plants. (A) Schematic representation of the domain structure of the Arabidopsis homolog of human U11-48K. The conserved CHHC-type zinc finger (ZF) motif and arginine (Arg)-rich region are shown. (B) GFP signals from the 48K–GFP-expressing tobacco plant were observed using a confocal microscope. DAPI was used to stain the nucleus. Scale bar=10 μm. (C) Position of the artificial miRNA1 (amiR1) target site and the sequences of amiR1, along with its target, U11-48K (48K). Exons and introns are represented as gray boxes and thick lines, respectively, and the untranslated regions are represented as white boxes. (D) Confirmation of mature *amiR1* generation. Total RNA extracted from each transgenic line (amiR1-1, amiR1-2, and amiR1-3) was separated via denaturing 12% PAGE, and the expression of 21 nucleotide long mature *amiR1* in each line was confirmed by northern blotting. (E, F) Down-regulation of *U11-48K* in the transgenic plants. The levels of *U11-48K* in each transgenic plant were confirmed by (E) RT–PCR and (F) real-time RT–PCR analysis. The numbers 1, 2, and 3 in (F) indicate amiR1-1, amiR1-2, and amiR1-3, respectively. Values are means ±SE obtained from three independent biological replicates. (This figure is available in colour at *JXB* online.)

*Arabidopsis thaliana* Columbia-0 ecotype, *u11/u12-31k* mutant (Kim *et al.*, 2010), *u11/u12-65k* mutant (Jung and Kang, 2014), and *u11-48k* mutant (this study) plants were grown at 23 °C under long-day conditions (16h light/8h dark cycle) either in soil or in half-strength Murashige and Skoog medium containing 1% sucrose. For the construction of artificial miRNA (amiRNA)-mediated mutant plants, two amiRNAs with different target sites (Fig. 1A; Supplementary Fig. S3A at *JXB* online) were designed using the Web MicroRNA Designer (http://wmd3.weigelworld.org/) as previously described (Kim *et al.*, 2010). The amiRNAs targeting the U11-48K gene were cloned into the pBI121 vector that expresses amiRNA under the control of the *Cauliflower mosaic virus* 35S promoter. The pBI121 vector was transformed into Arabidopsis by vacuum infiltration (Bechtold and Pelletier, 1998) using *Agrobacterium tumefaciens* GV3101. The T_3_ homozygous lines were selected and used for phenotype analysis.

### Analysis of cellular localization of the Arabidopsis homolog of human U11-48K

To determine the cellular localization of the Arabidopsis homolog of human U11-48K protein, the cDNA encoding a full-length U11-48K was amplified with gene-specific primers (forward primer, 5' ATGGATCGACCACCGTCGTTG 3'; and reverse primer, 5' TCACTCTTTTTCTGTTGGTATG 3'), and ligated in front of a green fluorescent protein (GFP) gene in the *Cassava vein mosaic virus* CsV-GFP3-PA vector, which expresses the fusion protein under the control of the CsV promoter, and the resulting vector was infiltrated into tobacco leaves using *A. tumefaciens* GV3101. The GFP signals in the leaves of the tobacco plants were observed using a Zeiss LSM510 laser scanning confocal microscope (Carl Zeiss, *http://www.zeiss.com/microscopy/*) with the excitation and emission wavelengths of 488nm and 505nm, respectively. The nuclei were stained with DAPI (4',6-diamidino-2-phenylindole), and the signals were observed with the excitation and emission wavelengths of 360nm and 460nm, respectively.

### Reverse transcription–PCR (RT–PCR) and northern blot analysis

To determine the expression levels of target genes, total RNA was extracted from the frozen tissues using the Plant RNeasy extraction kit (Qiagen, http://www.qiagen.com), and 200ng of RNA was reverse transcribed and amplified using the One-step RT-PCR kit (Qiagen) with the gene-specific primers listed in Supplementary Table S1. To detect 21-mer mature amiRNAs in the transgenic plants, 20 μg of total RNA was separated via denaturing 12% PAGE and transferred to a nylon membrane, as previously described (Kim *et al.*, 2010; Jung and Kang, 2014). RNA blots were hybridized with a radiolabeled probe complementary to the amiRNA, and the signals were detected using a FLA7000 Phosphorimager (GE Healthcare Life Sciences, http://www.gelifesciences.com).

### Analysis of the splicing patterns of U12 intron-containing genes

Total RNA was extracted from 3-week-old wild-type and amiRNA mutant plants. The RNA samples were treated with RQ1 DNase (Promega, http://www.promega.com) and further purified using an RNeasy clean-up kit (Qiagen). RT–PCR analysis of the splicing patterns was conducted essentially as described previously (Kim *et al.*, 2010; Jung and Kang, 2014). Briefly, 200ng of RNA was amplified using a One-step RT-PCR kit (Qiagen) with gene-specific primers that were designed to form base pairs with the 5' end of the first exon and the 3' end of the last exon in each gene (Kim *et al*, 2010; Supplementary Table S1). The PCR products were separated on a 1–2% agarose gel and visualized under UV light. The identities of the PCR products were verified by cloning and sequencing.

### Quantitative analysis of splicing efficiency

The splicing efficiency of U12 introns was analyzed by quantitative real-time RT–PCR as described (Koprivova *et al.*, 2010; Gu *et al.*, 2014). The gene-specific oligonucleotide primers corresponding to the exon/exon regions were designed to amplify the spliced (mature) transcripts, and the primers corresponding to the intron/exon regions were designed to amplify the unspliced (precursor) transcripts (Supplementary Table S2). A 100ng aliquot of total RNA was analyzed using a QuantiTect SYBR RT-PCR kit (Qiagen) with the gene-specific primers in a Rotor-Gene Q real-time thermal cycling system (Qiagen).

## Results

### Structural features and characterization of Arabidopsis homolog of human U11-48K

U11-48K proteins harbor a CHHC-type zinc finger motif at the N-terminal half and an arginine-rich region at the C-terminal half (Fig. 1A). Arabidopsis homolog of human U11-48K protein is comprised of 714 amino acids, in contrast to the human U11-48K protein which contains only 339 amino acids (Supplementary Table S3). A large difference in the length of the U11-48K proteins in human and Arabidopsis has already been documented, which demonstrates that the difference is mainly due to a long amino acid extension at the N-terminus of the Arabidopsis protein (Lorkovic *et al.*, 2005). The previous report also demonstrated that although low sequence homology was observed between the 48K proteins, conserved amino acids were found throughout the entire length of the protein, indicating that they are true orthologs of the U11-48K proteins (Lorkovic *et al.*, 2005). Here, we analyzed in more detail structural features of plant U11-48K proteins. Unlike animal U11-48K proteins that are comprised of 330–340 amino acids and share ~90% amino acid sequence similarity with each other, the length and amino acid sequences of plant U11-48K proteins tend to be more diverse (Supplementary Table S3). Analysis of the amino acid sequences revealed that U11-48K proteins in dicotyledonous plants contained ~700 amino acids, whereas those in monocotyledonous plants were comprised of ~400–600 amino acids (Supplementary Table S3). Sequence comparison indicated that the sequence similarity of U11-48K proteins among dicot plants was ~30–80%, whereas monocot plants share ~50–90% similarity (Supplementary Table S3). Comparison between dicots and monocots revealed amino acid sequence similarity of ~30%, while the similarities between plants and humans were <25% (Supplementary Table S3). Notably, the length and amino acid sequences of U11-48K proteins were more diverse than those of other plant U11/U12 proteins, which share ~60% sequence similarity among dicot and monocot plant species (Supplementary Table S3). Although diverse sequence similarity was observed for the entire peptide of the U11-48K proteins, all harbored a well-conserved CX5HX9HX3C-type zinc finger motif at the N-terminal half and an arginine-rich region at the C-terminal half (Supplementary Fig. S1). Importantly, the human U11-48K protein also harbors a CX5HX9HX3C-type zinc finger motif at the N-terminal half, which is necessary for the specific binding to the 5' splice site of U12-type introns (Tidow *et al.*, 2009). Our present analysis and previous reports indicate that plant U11-48K proteins are true orthologs of the U11-48K proteins. However, considering the lack of direct experimental evidence to support the binding of plant U11-48K protein to the 5' splice site of U12 introns, we named it Arabidopsis homolog of human U11-48K. To determine the subcellular localization of the Arabidopsis homolog of human U11-48K, the vector expressing the 48K–GFP fusion protein was transiently expressed in tobacco leaves, and the cellular expression of the 48K–GFP fusion protein was examined via confocal analysis. The results showed that the GFP signals were exclusively observed in the nucleus (Fig. 1B), indicating that Arabidopsis homolog of human U11-48K protein is localized to the nucleus.

### Arabidopsis homolog of human U11-48K plays an essential role in the normal growth and development of Arabidopsis

To determine the functional roles of Arabidopsis homolog of human U11-48K during plant growth and development, we first tried to obtain homozygous T-DNA insertion mutant lines. Two mutant lines, SALK_142557C and SALK_106445C in which the T-DNA was inserted into the first exon and the fourth exon of the U11-48K gene, respectively, were obtained from the Arabidopsis Biological Resource Center and were analyzed. However, we were not able to select the homozygous allele of the mutants, suggesting that Arabidopsis homolog of human U11-48K may play an essential role in plant development. We therefore generated *u11-48k* knockdown mutant plants using the amiRNA-mediated knockdown method, as described previously (Schwab *et al.*, 2006; Kim *et al.*, 2010). Three amiRNA lines targeted to the first exon in the *U11-48K* gene were generated (Fig. 1C) and used for phenotype analysis. Expression of the mature 21 nucleotide long amiRNA and down-regulation of *U11-48K* in the mutant plants were confirmed by northern blot and RT–PCR analyses (Fig. 1D–F).

**Fig. 2.**
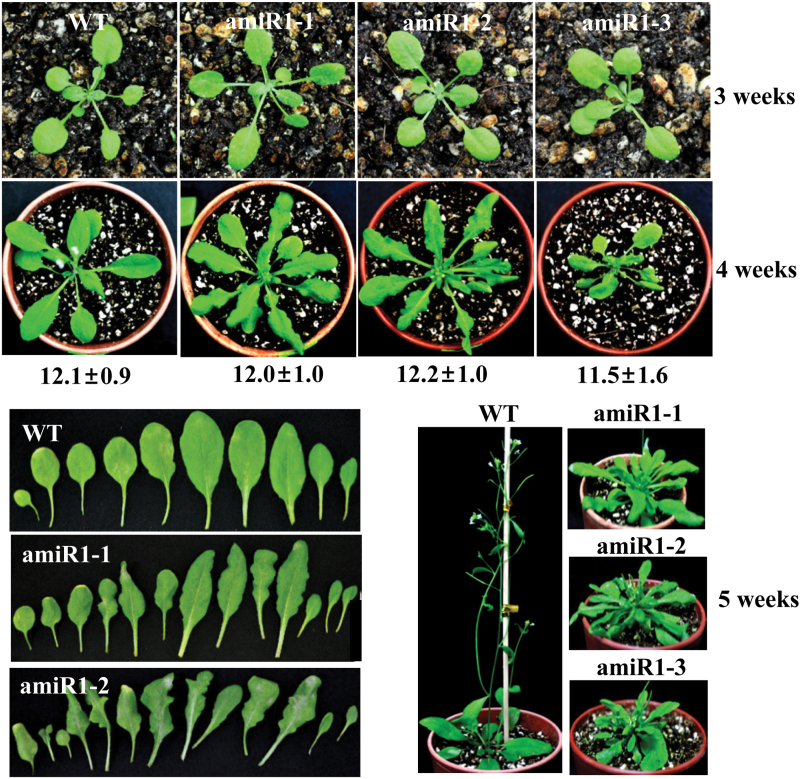
Development-defect phenotypes of the *u11-48k* mutant plants. The wild-type (WT) and artificial miRNA-mediated mutant (amiR1-1, amiR1-2, and amiR1-3) plants were grown in soil under long-day conditions, and photographs were taken on the indicated weeks. Identical results were obtained from three independent biological replicates, for which a representative example is shown. The numbers below the 4 week panel indicate the number of leaves present when floral buds appeared. Values are means ±SE obtained from three independent biological replicates, five plants per replicate. (This figure is available in colour at *JXB* online.)

The wild-type and *u11-48k* mutant plants displayed similar growth with no noticeable differences during the first 3 weeks (Fig. 2). However, at a later stage of growth, the *u11-48k* mutant plants exhibited several defects in growth and development, such as severely arrested primary inflorescence stems, formation of serrated leaves, and production of many rosette leaves after bolting (Fig. 2). Since the *u11-48k* mutants exhibited severe defects in the formation of primary inflorescence stems, we carefully examined whether Arabidopsis homolog of human U11-48K is involved in flowering time control. However, no differences in leaf numbers were observed between the wild type and *u11-48k* mutants at the time of flowering (Fig. 2), indicating that flowering time is not affected by Arabidopsis homolog of human U11-48K. To confirm further whether the developmental-defect phenotypes observed in the *u11-48k* mutants resulted from the specific knockdown of the *U11-48K* gene, another amiRNA line targeted to a different position in the first exon of the *U11-48K* gene was generated, and their phenotypes were analyzed. Identical developmental-defect phenotypes were observed in the second amiR2 mutant line, comparable with the first amiR1 mutant line (Supplementary Fig. S2). Altogether, these results clearly indicate that Arabidopsis homolog of human U11-48K plays an essential role in the normal growth and development of Arabidopsis.

### Arabidopsis homolog of human U11-48K plays a role in senescence

**Fig. 3.**
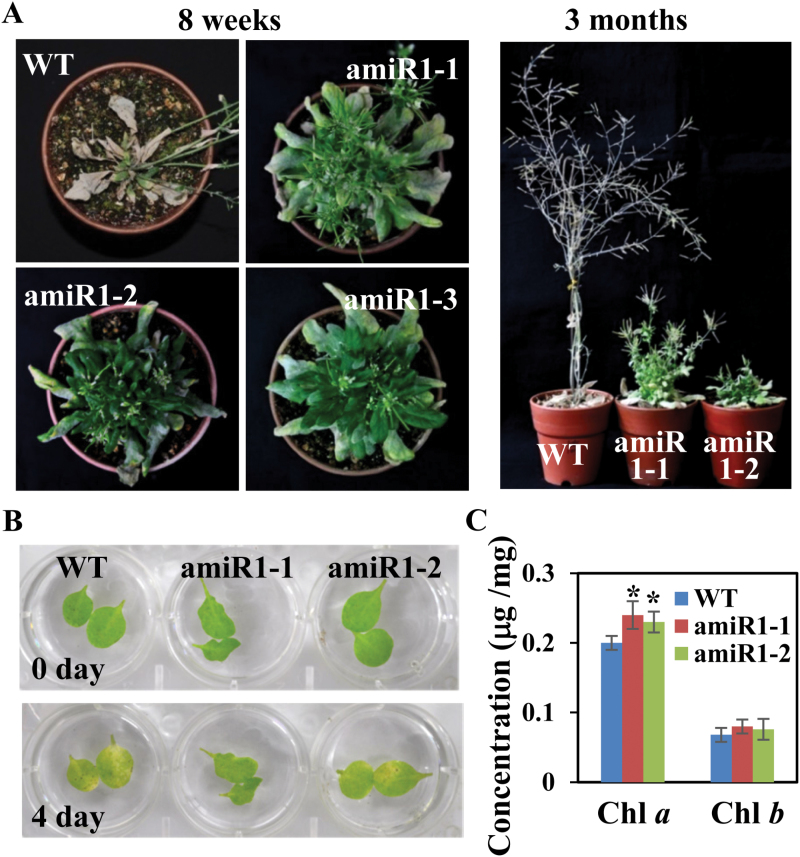
Delayed senescence of the *u11-48k* mutant plants. (A) Prolonged survival of the *u11-48k* mutant plants (amiR1-1, amiR1-2, and amiR1-3). Identical results were obtained from three independent experiments, for which a representative example is shown. (B) Dark-induced senescence. Rosette leaves from 4-week-old wild type (WT) and *u11-48k* mutants were floated on water in the dark for 4 d. (C) Chlorophyll content in the leaves of each plant was measured 4 d after dark-induced senescence. Values are means ±SE obtained from three independent biological replicates, two leaves per replicate, and asterisks above the columns indicate statistically different values between WT and mutant plants (*P*≤0.05). (This figure is available in colour at *JXB* online.)

As it was evident that Arabidopsis homolog of human U11-48K is involved in the growth and development of Arabidopsis, we subsequently examined whether it is involved in senescence. When the plants were grown in soil for >8 weeks, the *u11-48k* mutants survived beyond the death of the wild-type plants (Fig. 3A; Supplementary Fig. S2). To examine further the possible involvement of Arabidopsis homolog of human U11-48K in senescence, the dark-induced senescence assay was performed on the leaves of 4-week-old plants. The results showed that greening of the leaves of *u11-48k* mutant plants was maintained for a much longer period than in the wild-type plants when incubated under dark conditions (Fig. 3B). Consequently, the Chl *a* contents in the *u11-48k* mutant plants were higher than those in the wild-type plants (Fig. 3C). These results suggest that Arabidopsis homolog of human U11-48K is positively involved in senescence.

### Exogenously applied gibberellic acid partially recovers the stunted stem growth in the *u11-48k* mutant plants

**Fig. 4.**
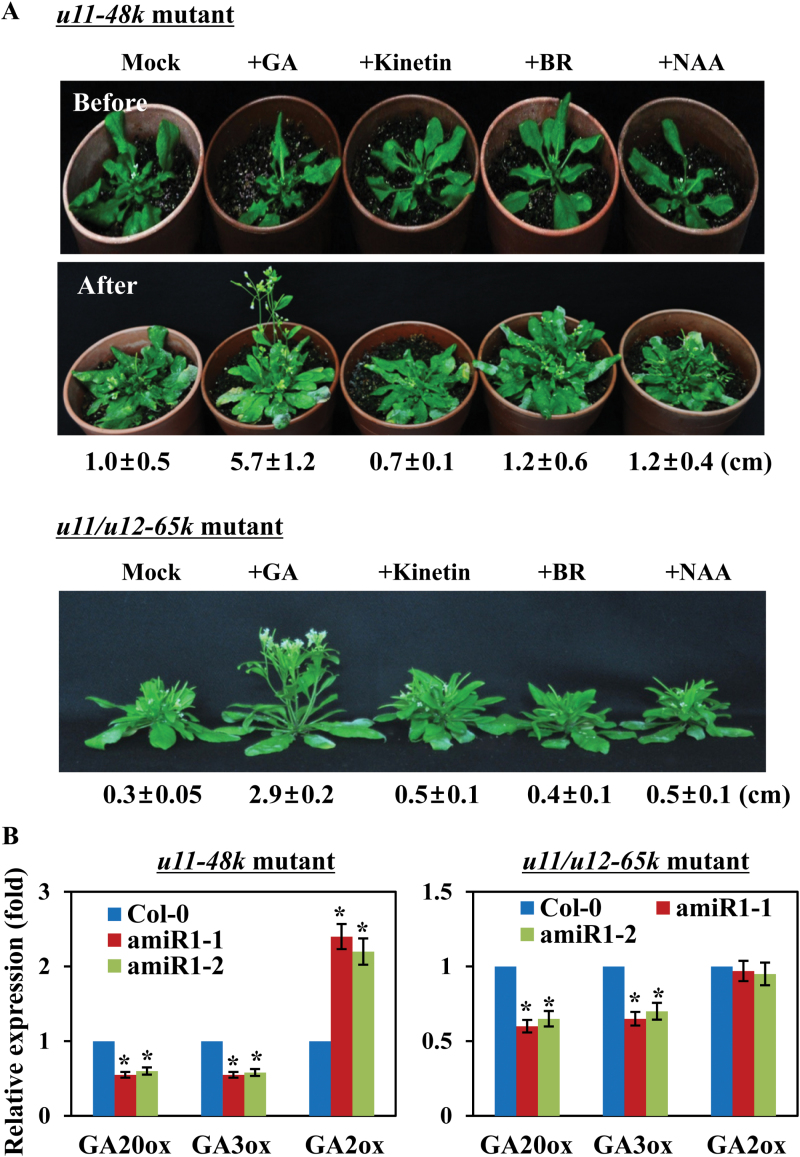
Effect of exogenously applied hormones on the stem length of the *u11-48k* and *u11/u12-65k* mutant plants. (A) Four-week-old mutant plants were treated with 100 µM GA, 50 µM kinetin, 5 µM BR, or 0.5 µg ml^–1^ NAA, and stem lengths of the plants were measured 2 weeks after the application of each hormone. Values are means ±SE obtained from three independent experiments, five plants per experiment. (B) The transcript levels of the genes involved in GA metabolism were determined in the meristemic region of the mutant plants. Values are means ±SE obtained from three independent biological replicates, and asterisks above the columns indicate statistically different values between wild-type and mutant plants (*P*≤0.05). (This figure is available in colour at *JXB* online.)

A previous study demonstrated that the stunted inflorescence stems of *u11/u12-31k* mutants were recovered by the application of gibberellic acid (GA) (Kim *et al.*, 2010). To determine whether the phenotype of stunted primary inflorescence stems was related to hormone functions, the effect of exogenously applied hormones on the growth of the *u11-48k* mutant and *u11/u12-65k* mutant plants (Jung and Kang, 2014) was next examined. To accomplish this, the amiR1 mutant plants were treated with different hormones, including GA_3_ (100 µM), cytokinin (kinetin, 50 µM), brassinosteroid [BR (24-epibrassinolide, 5 µM], and auxin [α-naphthalene acetic acid (NAA), 0.5 µg ml^–1^], all of which are known to influence stem growth and development, and then the growth of primary inflorescence stems was examined. The results showed that the length of the primary inflorescence stems of the *u11-48k* and *u11/u12-65k* mutant plants had increased by ~6-fold (from 1.0cm to 5.7cm) and 10-fold (from 0.3cm to 2.9cm) 2 weeks after GA application, respectively, whereas none of the other hormones affected the growth of the primary inflorescence stems of the mutant plant (Fig. 4A). To support that the GA-mediated recovery of the stem growth is specific to the *u11-48k* and *u11/u12-65k* mutant plants, the growth of the primary inflorescence stems of the wild-type plants was examined after application of each hormone. Notably, none of the hormones affected the growth of the primary inflorescence stems of the wild-type plants (Supplementary Fig. S3). These results suggest that the abnormal growth and stunted inflorescence stems of the *u11-48k* and *u11/u12-65k* mutants as well as the *u11/u12-31k* mutants are partially related to the defect in GA biosynthesis.

Because the inflorescence stem growth of the *u11-48k* and *u11/u12-65k* mutants is partially recovered by exogenously applied GA, we next determined whether the genes involved in GA metabolism are disturbed in the knockdown plants. We investigated, via real-time RT–PCR analysis using the primers listed in Supplementary Table S2, the expression levels of the genes encoding key enzymes involved in GA metabolism: GA20-oxidase (GA20ox) and GA3-oxidase (GA3ox) involved in GA biosynthesis, and GA2-oxidase (GA2ox) involved in GA catabolism. Because exogenously applied GA influences the growth of primary inflorescence stems, we specifically wanted to determine the expression levels of these genes in meristemic regions of the plants. When the *u11-48k* and *u11/u12-65k* mutant plants began to bolt, the stems, leaves, and roots of the plants were removed and only the meristem-containing regions were collected for RNA extraction and subsequent analysis. Notably, the transcript levels of the genes involved in GA biosynthesis (*GA20ox* and *GA3ox*) were significantly down-regulated in the *u11-48k* and *u11/u12-65k* mutants compared with the wild-type plants (Fig. 4B). The transcript level of the gene involved in GA catabolism (*GA2ox*) was markedly up-regulated in the *u11-48k* mutants, but was not noticeably reduced in the *u11/u12-65k* mutants (Fig. 4B). A large decrease in the transcript levels of GA biosynthesis-related genes together with a significant increase in the transcript level of the GA catabolism-related gene in the *u11-48k* mutants and a marked decrease in the transcript levels of GA biosynthesis-related genes in the *u11/u12-65k* mutants suggest that the amounts of GA in both mutants may be reduced compared with those in the wild-type plants, which is consistent with the observation that exogenously applied GA partially recovers the stunted stem growth in both mutant plants (Fig. 4A). To examine whether the genes encoding GA20ox, GA3ox, and GA2ox contain U12 introns, and if their splicing patterns are altered in the mutant plants, the intron sequences in these GA metabolism-related genes were analyzed. None of the *GA20ox, GA3ox*, and *GA2ox* genes contained U12 introns (the U12-type intron database; http://genome.crg.es/cgi-bin/u12db/u12db.cgi; Supplementary Fig. S4), and splicing patterns of *GA20ox, GA3ox*, and *GA2ox* genes were not altered in the mutants compared with those of the wild-type plants (Supplementary Fig. S4). These results suggest that the defect in GA biosynthesis is responsible, at least in part, for abnormal growth and stunted inflorescence stems of the *u11-48k* and *u11/u12-65k* knockdown plants.

### Arabidopsis homolog of human U11-48K is essential for correct splicing of U12 introns

**Fig. 5.**
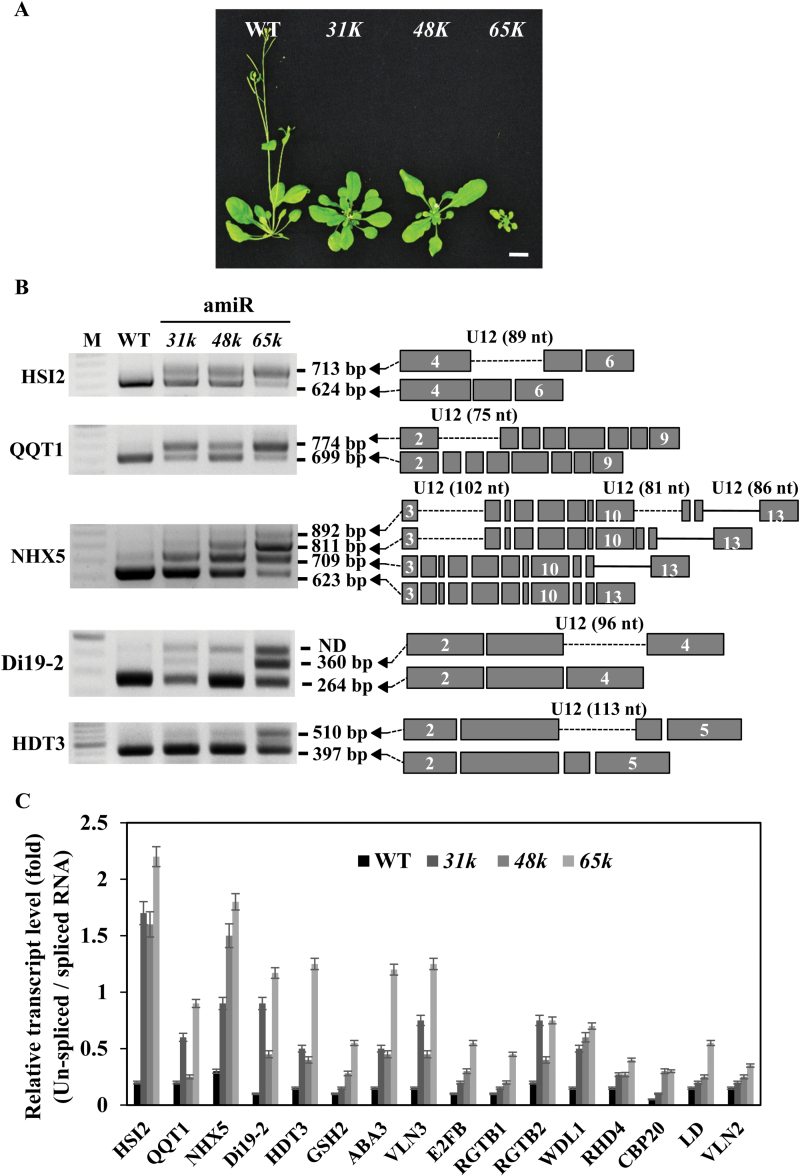
Comparison of phenotype and abnormal splicing of U12 introns in the *u11/u12-31k*, *u11-48k*, and *u11/u12-65k* mutant plants. (A) Growth-defect phenotypes of the *u11/u12-31k* (*31k*), *u11-48k* (*48k*), and *u11/u12-65k* (*65k*) mutant plants. Scale bar=1cm. (B) The splicing patterns of selected U12 intron-containing transcripts were analyzed by RT–PCR in wild type (WT), *31k*, *48k*, and *65k* mutant plants. All primers used were designed to form base pairs with the 5' end of the first exon and the 3' end of the last exon in each gene. Identical results were obtained from three independent experiments, for which a representative example is shown. (C) Relative levels of spliced and unspliced transcripts of U12 intron-containing genes in the *u11/u12-31k*, *u11-48k*, and *u11/u12-65k* mutant plants were analyzed by quantitative real-time RT–PCR, and the levels of the unspliced forms relative to those of the spliced forms are expressed in each genotype. Data are the means ±SE obtained from three independent biological replicates. (This figure is available in colour at *JXB* online.)

To ascertain whether the abnormal growth and development of the *u11-48k* mutant plants were due to defects in the splicing of U12-type introns, the splicing patterns of U12-type introns were investigated in the *u11-48k* mutants at the time of bolting. Arabidopsis harbors ~230 U12-type intron-containing genes (the U12-type intron database; http://genome.crg.es/cgi-bin/u12db/u12db.cgi; Jung and Kang, 2014). Among them, those known to be conserved across organisms and whose putative functions have been clearly identified were chosen, and their splicing patterns were analyzed by RT–PCR as previously described (Kim *et al.*, 2010; Jung and Kang, 2014). The splicing of all U12 introns examined in this study, including HIGH-LEVEL EXPRESSION OF SUGAR-INDUCIBLE GENE 2 (HSI2), Quartre-QuarT 1 (QQT1), NHX5, NHX6, GSH2, RGTB2, and drought induced 19-2 (Di19-2), was found to be defective in the *u11-48k* mutant plants (Fig. 5; Supplementary Fig. S5). These results indicate that the developmental defects observed in *u11-48k* mutant plants were due to impairment in U12 intron splicing.

### The severity of developmental-defect phenotypes of the mutants is closely related to the degree of impairment in U12 intron splicing

Given the essential roles of U11/U12-31K (Kim *et al.*, 2010), U11/U12-65K (Jung and Kang, 2014), and Arabidopsis homolog of human U11-48K (this study) in U12 intron splicing as well as normal growth and development of plants, it would be interesting to determine whether the severity of developmental-defect phenotypes of each mutant is related to the degree of impairment in U12 intron splicing. To address this question, the splicing patterns of the U12 introns in *u11/u12-31k*, *u11-48k*, and *u11/u12-65k* mutant plants were analyzed in parallel. Growth of the *u11/u12-65k* mutant was observed to be more severely retarded than that of *u11/u12-31* and *u11-48k* mutants (Fig. 5A). Notably, splicing of all U12 introns analyzed was impaired only in the *u11/u12-65k* mutant, whereas splicing of several U12 introns examined in this study was not affected in *u11/u12-31k* and *u11-48k* mutants. Moreover, the degree of impairment in U12 intron splicing was indeed related to the severity of the developmental-defect phenotypes. The intensities of pre-mRNAs were much stronger in the *u11/u12-65k* mutant than in both the *u11/u12-31* and *u11-48k* mutants. Consequently, the intensities of mature mRNAs were much weaker in the *u11/u12-65k* mutant (Fig. 5B; Supplementary Fig. S6), demonstrating that the defects in U12 intron splicing were much more significant in the *u11/u12-65k* mutant than in the *u11/u12-31* and *u11-48k* mutants. To confirm further the differences in the defects in U12 intron splicing among different mutant plants, the levels of mature (spliced) and precursor (unspliced) RNAs were quantitatively determined by real-time RT–PCR analysis. The results showed that the relative levels of unspliced RNAs in the *u11/u12-65k* mutants were much higher than those in the *u11/u12-31* and *u11-48k* mutants (Fig. 5C). To verify that the mutation of U11/U12-31K, U11-48K, or U11/U12-65K does not affect the splicing of U2-type introns, the splicing patterns of selected U2 intron-containing transcripts were analyzed by RT–PCR in the mutant plants. Clearly, splicing of all U2 intron genes tested was not altered in the mutant plants (Supplementary Fig. S7), confirming that splicing of only U12-type introns was affected in the mutant plants. Altogether, these results indicate that the severity of developmental defects in the mutants is indeed closely correlated with the degree of impairment in U12 intron splicing, reinforcing the conclusion that the correct splicing of U12 introns is essential for the normal growth and development of plants.

## Discussion

The present study clearly demonstrated that Arabidopsis homolog of human U11-48K protein, the minor spliceosomal protein which interacts with the U11 snRNA and recognizes the 5' splice site of U12 introns in humans (Turunen *et al.*, 2008; Tidow *et al.*, 2009), is indispensable for correct U12 intron splicing, and crucial for the normal growth and development of plants. Similar to U11/U12-31K and U11/U12-65K (Kim *et al.*, 2010; Jung and Kang, 2014), Arabidopsis homolog of human U11-48K was observed to affect plant development after the bolting stage rather than during the vegetative stage (Fig. 2). The present finding of the essential role of Arabidopsis homolog of human U11-48K in plant development further reinforces the previously determined roles of the minor spliceosomal proteins in cell proliferation and viability of plants, as well as in animals. As U12 introns are often found in genes involved in information processes, such as DNA repair, RNA processing, and translation (Burge *et al.*, 1998; Otake *et al.*, 2002; Will *et al.*, 2004; König *et al.*, 2007), it is clear that tight regulation of U12 intron splicing by Arabidopsis homolog of human U11-48K, as well as U11/U12-31K and U11/U12-65K, is essential for the normal development of living organisms which harbor U12-type introns.

Despite the increasing amount of information on the role of Arabidopsis homolog of human U11-48K, U11/U12-31K, and U11/U12-65K in U12 intron splicing and plant growth and development, it remains unknown at present whether the observed developmental defects in these mutant plants were caused by the abortion of splicing of all U12-type introns. It is difficult to interpret the importance and exact role of U12 intron-containing genes in plant growth and development due to the fact that >50 of the putative U12 intron-containing genes encode proteins whose biological and molecular functions are as yet unknown. In addition, as previously observed in *u11/u12-65k* mutant plants, defects in U12 intron splicing affect diverse alternative splicing, including exon skipping, intron retention, alternative 3' sites, and alternative 5' sites (Jung and Kang, 2014). Although it is still premature to link the observed development-defect phenotypes directly to impairment in the splicing of specific U12 introns, it is likely that cumulative defects in the splicing of growth- and development-related U12 intron-containing genes, such as POD, which regulates early embryo patterning (Li *et al.*, 2011), QQT1, which is associated with microtubules during cell division (Lahmy *et al.*, 2007), HSI2, which is involved in seedling growth (Tsukagoshi *et al.*, 2007), and the Di19 gene family, which is involved in stress and light signaling pathways (Milla *et al.*, 2006), are responsible for the abnormal development phenotypes observed in these mutant plants. Notably, the comparative analysis carried out herein on the splicing defects and abnormal development phenotypes among the *u11/u12-31k, u11-48k*, and *u11/12-65k* mutants revealed that the severity of abnormal development was closely correlated with the degree of impairment in U12 intron splicing (Fig. 5). These results reinforce the proposition that cumulative defects in the splicing of U12 intron-containing genes result in abnormal growth and development of the mutant plants.

The process of intron splicing occurs through spliceosome assembly, splicing, and subsequent spliceosome disassembly (Staley and Guthrie, 1998, 1999), during which interactions between spliceosome-associated proteins and snRNAs are required. It has been demonstrated from studies on animal systems that U11-48K protein interacts with the U11 snRNA and recognizes the 5' splice site of U12 introns (Turunen *et al.*, 2008; Tidow *et al.*, 2009). The U11/U12-65K protein was shown to interact with U12 snRNA and recognize the branch-point sites of U12 introns during minor spliceosome assembly in humans and Arabidopsis (Benecke *et al.*, 2005; Jung and Kang, 2014). As a key component of the core complexes between U11/U12-65K, -59K and -48K proteins found in plants and animals (Tidow *et al.*, 2009; Jung and Kang, 2014), the U11-48K protein is necessary for spliceosome assembly and U12 intron splicing in plants, as well as in animals.

In conclusion, our findings clearly demonstrate that Arabidopsis homolog of human U11-48K is an indispensable spliceosomal protein, crucial for U12 intron splicing and the normal growth and development of plants. As evidenced by the close correlation of the severity of abnormal development among the *u11/u12-31k*, *u11-48k*, and *u11/12-65k* mutants with the degree of impairment in U12 intron splicing, it is likely that cumulative defects in the splicing of U12 intron-containing genes resulted in the abnormal growth and development observed in mutant plants. Future research should focus on analyzing whether the observed development-defect phenotypes are due to the impairment in the splicing of specific U12 introns or a subset of U12 introns in cells.

## Supplementary data

Supplementary data are available at *JXB* online.

Figure S1. Amino acid sequence comparison of U11-48K proteins in diverse plant species.

Figure S2. Development-defect phenotypes of the *u11-48k* mutant plants.

Figure S3. Effect of exogenously applied hormones on the stem length of the wild-type plants.

Figure S4. Splicing of *GA20ox, GA3ox*, and *GA2ox* genes in the mutant plants.

Figure S5. Abnormal splicing of U12 introns in the *u11-48k* mutant plants.

Figure S6. Comparison of abnormal splicing of U12 introns in the *u11/u12-31k*, *u11-48k*, and *u11/u12-65k* mutant plants.

Figure S7. Splicing of selected U2 introns in the mutant plants.

**Table S1.** Gene-specific primer pairs used in RT–PCR experiments.

**Table S2.** Gene-specific primer pairs used in quantitative analysis of splicing efficiency by real-time RT–PCR.

**Table S3.** Comparison of U11-48K proteins and other U11/U12 proteins found in diverse plant species and in humans.

Supplementary Data
