# Supplementary material for: The Arabidopsis homolog of human minor spliceosomal protein U11-48K plays a crucial role in U12 intron splicing and plant development
Source: J Exp Bot. 2016 Apr 17;67(11):3397–406. doi: 10.1093/jxb/erw158 (PMC4892727; doi:10.1093/jxb/erw158)
Supplement: Supplementary Data [file supp_67_11_3397__index.html]

The Arabidopsis homolog of human minor spliceosomal protein U11-48K plays a crucial role in U12 intron splicing and plant development — The Arabidopsis homolog of human minor spliceosomal protein U11-48K plays a crucial role in U12 intron splicing and plant development — Supplementary Data 

# The Arabidopsis homolog of human minor spliceosomal protein U11-48K plays a crucial role in U12 intron splicing and plant development

## Supplementary Data

Data files

- Supplemental\_tables\_S1\_S3\_Supplemental\_Figures\_S1\_S7.pdf - Supplementary Data
