# Supplementary material for: The Arabidopsis homolog of human minor spliceosomal protein U11-48K plays a crucial role in U12 intron splicing and plant development
Source: J Exp Bot. 2016 Apr 17;67(11):3397–406. doi: 10.1093/jxb/erw158 (PMC4892727; doi:10.1093/jxb/erw158)
Supplement: Supplementary Data [file supp_erw158_Supplemental_tables_S1_S3_Supplemental_Figures_S1_S7.pdf]

Supplementary Table S1. Gene-specific primer pairs used in RT-PCR experiments.

Gene	Primer (5’ to 3’)
For RT-PCR	
At3g04160 (U11-48K)	Forward: ATGGATCGACCACCGTCGTT<br>Reverse: TGGGCAACGAAGTGAATGAAGAA
At1g56280 (Di19-1)	Forward: CTTTAGAGTCGAAGAACGCGGTATGC<br>Reverse: GAGAACTCTGTGATCGTGTAGGTGAG
At1g02750 (Di19-2)	Forward: CGATATAGCAGTGGAGTATCCATGC<br>Reverse: GGCAACTCATCAAGATGAGGTTGAAG
At3g05700 (Di19-3)	Forward: GGGAAGAGTTTGCTTGCCCTTTCTG<br>Reverse: CCTCCAAAGAGGCTCTGAAAGTTTCC
At3g06760 (Di19-4)	Forward: CAGAGTTTATATGCCCTTTTTGCGCTG<br>Reverse: GAAGATCCACCAAGAAGAGACTGTAG
At2g30470 (HSI2)	Forward: GGTAAGCTAATGAGTCCAGAAAGTAC<br>Reverse: CATGGACTGTATGCATGGAGTGAC
At5g22370 (QQT1)	Forward: GAAGGTTGCTATTGTTAATCTGGATCC<br>Reverse: CTTGCATCAATGCCGGCAAATATG
At5g44200 (CBP20)	Forward: GAGAGGATACTGAAGATGCAGTCAAG<br>Reverse: CTTCTTGAGTTATCGCGGCCATAG
At1g54370 (NHX5)	Forward: CGTGGTTTAATTTCCACGAAGAGTTC<br>Reverse: GAAGAGTATAGAAACAATGCCGGAG
At1g79610 (NHX6)	Forward: GTTGGTGGTTTAGCGAACATCTCGAAC<br>Reverse: CGATACGATGCCAGATAGACTGAGAC
At2g36010 (E2FA/E2F3)	Forward: CATGCTTGCCTATTGCTTCTAGCTC<br>Reverse: CAACAGCTGTTGGTGCTATTTCGC
At5g22220 (E2FB/E2F1)	Forward: GTTCCTGATGAACCCTCTAATGTCC<br>Reverse: GTGATCTCGTAGCAGTGGATTCTTC
At5g03740 (HDT3)	Forward: GAATGGCAAACCACTTCACCTAG<br>Reverse: GTCATCATCTTGCTTGGCTTTGAC
At2g21880 (RabG2)	Forward: GGTCATTGTTCTTGGAGATAGCGG<br>Reverse: GAGGAACTCTGTGTGCCAGTTATTG
At1g16540 (ABA3/los5)	Forward: GATTATTATGGATACCCAGATGGTCCC<br>Reverse: GCATATTCCCTAATACCAAGTACACTG
At2g41740 (VLN2)	Forward: GATTTGACTGTTATACAATGTCAAC<br>Reverse: GCAAGTATATAACCGGGTCTC
At3g57410 (VLN3)	Forward: TAGCCACTAATACAAAATGTCTGG<br>Reverse: TCCTCCTCCGGTTTTTTAAATC
At3g04630 (WDL1)	Forward: TGCTTATGGACCGAAACGC<br>Reverse: TTCAAGCTCGTTCCTCTCAG
At5g12210 (RGTB1)	Forward: GCTGACAAGCATGTACGTTAC<br>Reverse: CAACGCAGCAGAAAATTTGTC
At3g12070 (RGTB2)	Forward: GAAGATGAAGTTGTGTCATGGG<br>Reverse: ATGAGACTCCGCTCCAGGTG
At3g51460 (RHD4)	Forward: AAGCTCTTAAGTGTAGCAGAAAAG<br>Reverse: CTTGTTAACGAGATCAACTGCC
At4g02560 (LD)	Forward: CGCAAATTGATTCGCAGAGTCAATTGC<br>Reverse: GAATATCATCAGCAGACATTGGGCG
At5g27380 (GSH2)	Forward: GATCAGATTGGCATAGACTCTGAACG<br>Reverse: GACGGATGATCATTAGGAGTATAGCC
Actin	Forward: CAGCAGAGCGGGAAATTGTAAGAG<br>Reverse: TTCCTTTCAGGTGGTGCAACGAC

For real-time RT-PCR

GA20ox	Forward: ACATGGGTTTCAGCCATTTGGGAAGGTGTA
	Reverse: AGATGGGTTTGGTGAGCCAATCTGAAAAGG
GA3ox	Forward: CCAACATCACCTCAACTACTG
	Reverse: CTCTTTCCATGTCACCGATTG
GA2ox	Forward: GTGTCGTACAAGGTGTTGGAGA
	Reverse: CCAAGTCTATAATCACCAAGC
Actin	Forward: TGCGATAATGGAACTGGTATGG
	Reverse: AAGACAGCCCTGGGCGCATCA

Supplementary Table S2. Gene-specific primer pairs used in quantitative analysis of splicing efficiency by real-time RT-PCR.

Gene		Primer (5' to 3')
HSI2	exon1	Forward: CCA ACA TGG TCC ATT GCT CT
	intron1	Reverse: CTG TCC AGA ATA AAA TAT TTG GC
	exon2	Reverse: CAG GCT TTT GGA AGA ACT AG
QQT1	exon1	Forward: GCC AAA TAT TTT ATT CTG GAC AG
	intron1	Reverse: CAA CAT ACA CCT GCA ATT TTT AAC
	exon2	Reverse: AAC ATC TTC TAA CTT GAT CAA TTC
NHX5	exon1	Forward: CTC CGC CGT CAT CGA TTC C
	intron1	Reverse: TAG CAG CAG CGA ATT TGA AG
	exon2	Reverse: GTA ACA ATG GCT CCA AAG TTA G
NHX6	exon1	Forward: CGG CGG CTC TGC GTT CTT T
	intron1	Reverse: CAA AAT TAG TAA GAG AAG TGC TC
	exon2	Reverse: AGT GAA CCT GTT AGT CCT CG
E2FA	exon1	Forward: CTC AGG ATC TAC AGG ACA CC
	intron1	Reverse: CTG CAC AAC TTC CTA TTT CTA TG
	exon2	Reverse: GTT TTC CAA ATA TCC GTC ATG C
E2FB	exon1	Forward: AAG CAC CGA AAG AAA CAT GG
	intron1	Reverse: AAG AGA GAT TAG ATT AAA TCA CAG
	exon2	Reverse: GTG GTC TTG ATC AAA TGT AAT C
HDT3	exon1	Forward: GTT TTG AGT GTG ACT GTT GG
	intron1	Reverse: TAT CTT CCA GTC TTA AAT ATA GAG
	exon2	Reverse: CAG GAG CAG GAA CAG CTT C
GSH2	exon1	Forward: GAT TGG CAT AGA CTC TGA AC
	intron1	Reverse: CAT AAG AAG TCA AGA TCG ACC
	exon2	Reverse: TCC TCT TGT ACA CTC CCT TC
LD	exon1	Forward: GTA TTG CTG AAA TCA TGG GTG
	intron1	Reverse: GTT AAC TTA TCC AAT GCA TTG AG
	exon2	Reverse: TGC GGA CTC TTT CCA GCA G
ABA3	exon1	Forward: AGA TGG TCC CAA GAA CAT TC
	intron1	Reverse: CAT TAA AAT CTG AGA TTG AAA GCC
	exon2	Reverse: GAT GCA TTA AAG TAT TCA AGC AC
Di19-1	exon1	Forward: CTT TAG AGT CGA AGA ACG CG
	intron1	Reverse: GAA TAT CTG TAA GTT ATA AAC GTA G
	exon2	Reverse: CAA CAG AAC CAT TAC GAG AAG
Di19-2	exon1	Forward: TAT GAT TTA GTT GAA TTG TGT CAC
	intron1	Reverse: AAT CAC ATT CAT TAT AGC GAC GG
	exon2	Reverse: GCT GAT GAT GAT TCA TAG ATG G
CBP20	exon1	Forward: TCC TGT TCT ACT CTA GAG AG
	intron1	Reverse: TTA ATT GTA AAG AAA CGG TCA GG
	exon2	Reverse: GCT GCC TTT GTG CTT CGA G

Supplementary Table S2. (Continued)

Gene		Primer (5’ to 3’)
RGTB1	exon1	Forward: TGG CTT TGC CGG TAA CAC TG
	intron1	Reverse: GAT TAA TTA ACA TAT GAA AGT TCC AC
	exon2	Reverse: ACA TTT CAA TAT TGA GAG ACA GC
RGTB2	exon1	Forward: GTG GCT TTG CCG GTA ATA CT
	intron1	Reverse: ACA ACA AAC GAA TTA CCG AG
	exon2	Reverse: ATA TTG AGA GGC AGC ATA TAG C
RHD4	exon1	Forward: GAG CTC ACA ACG CTT GCA TG
	intron1	Reverse: ACG TAA CCA TGA AGC GAA AAT AG
	exon2	Reverse: TGT ACC GTT TCT CCT AGT GC
VLN2	exon1	Forward: GTT CAT TGG CGT TTG TAG TAC
	intron1	Reverse: AAC AGT CCA AAC ATT CAG GAA G
	exon2	Reverse: CAC CCC CTT TAT TCT GTG TTG
VLN3	exon1	Forward: CTC TTG AAG GAA GAG ATT AGC
	intron1	Reverse: AAC ATT ACC AGC AAA CTG GTG
	exon2	Reverse: CAA ATA GAT AAG CGC CAC CC
WDL1	exon1	Forward: ATG CAC CAA ATG TTG GAT CTG
	intron1	Reverse: TAA ACA TCC ATA CAT AAG TAT CAA G
	exon2	Reverse: CAC TCC TAA ACG TTG GAG C

Supplementary Table S3. Comparison of U11-48K proteins and other U11/U12-proteins found in diverse plant species and humans.

48K	Species	Accession No.	aa*	Sequence homology (%)**									
				1	2	3	4	5	6	7	8	9	10
Dicots	*A. thaliana*	At3g04160	714		75	77	40	33	27	33	29	28	24
	E. salsugineum	XP_006408216	733			78	39	33	27	32	28	31	23
	C. rubella	XP_006297086	703				41	31	27	32	30	31	24
	R. communis	XP_002525479	722					35	29	32	30	31	20
	G. max	XP_003535384	687						30	32	30	32	19
Monocots	*H. vulgare*	BAJ90436	656							58	57	91	10
	S. bicolor	XP_002466198	481								85	56	13
	Z. mays	NP_001145685	527									55	12
	T. urartu	EMS45165	388										3
Humans	*H. sapiens*	NP_689764	339										

	Number of amino acids		Sequence homology (%)
	Arabidopsis	Other dicot and monocot plants	
65K	442	441~467	55~92
59K	382	384~420	55~93
35K	333	314~403	60~95
31K	261	261~275	60~95
25K	165	155~187	60~95

*Number of amino acid (aa)
**The sequence homology was calculated using the ClustalW2 program.

Supplementary Fig. S1 Amino acid sequence comparison of U11-48K proteins in diverse plant species. The alignment was made using the ClustalW2 program. Gaps in the sequences are indicated by dashes. The position of CHHC-type zinc finger and argine-rich region is indicated by a box and a thick line, respectively. Accession numbers of 48K-related proteins are as follow; *A. thaliana* (At3g04160), *R. communis* (XP_002525479), *C. clementina* (XP_006443313), *E. salsugineum* (XP_006408216), *C. rubella* (XP_006297086), *G. max* (XP_003535384), and *M. truncatula* (XP_003590902).

Supplementary Fig. S2 Development-defect phenotypes of the *u11-48k* mutant plants. (A) Schematic presentation and the sequences of artificial miRNA2 (amiR2) along with its target U11-48K (48K). Exons and introns are represented as gray boxes and thick lines, respectively. Downregulation of *U11-48K* in the transgenic plants was confirmed by RT-PCR and real-time RT-PCR analysis. (B) Development-defect phenotypes of the *u11-48k* knockdown plants. The wild-type (WT) and artificial miRNA-mediated knockdown mutant (amiR2) plants were grown in soil for the indicated times, and the abnormal leaf shape, arrested stem growth, and delayed senescence of the *u11-48K* mutant plants are shown. Identical results were obtained from three independent experiments, for which a representative example is shown.

Supplementary Fig. S3 Effect of exogenously applied hormones on the stem length of the wild-type plants. Twenty four-day-old Col-0 plants were treated with 100 μM GA, 50 μM kinetin, 5 μM BR, or 0.5 μg/ml NAA, and stem lengths of the plants were measured 2 weeks after the application of each hormone. Values are means ± SE obtained from three independent experiments (n=5).

Supplementary Fig. S4 Splicing of *GA20ox*, *GA3ox*, and *GA2ox* genes in the mutant plants. The splicing patterns of *GA20ox*, *GA3ox*, and *GA2ox* transcripts were analyzed by RT-PCR in wild type (WT), *48k*, and *65k* mutant plants. Exons and introns are represented as gray boxes and thin lines, respectively, and the positions of primers used are indicated by thick lines. The sequences of primer sets are shown. Identical results were obtained from three independent experiments, one of which is shown.

Supplementary Fig. S5 Abnormal splicing of U12 introns in the *u11-48k* mutant plants. The splicing patterns of selected U12 intron-containing transcripts were analyzed by RT-PCR in wild type (WT) and three different knockdown plants (amiR1-1, amiR1-2, and amiR1-3). Identical results were obtained from three independent experiments, one of which is shown.

Supplementary Fig. S6 Comparison of abnormal splicing of U12 introns in the *u11/u12-31k*, *u11-48k*, and *u11/u12-65k* mutant plants. The splicing patterns of selected U12 intron-containing transcripts were analyzed by RT-PCR in wild type (WT), *31k*, *48k*, and *65k* mutant plants. Identical results were obtained from three independent experiments, one of which is shown.

Supplementary Fig. S7 Splicing of selected U2 introns in the mutant plants. The splicing patterns of selected U2 intron-containing transcripts were analyzed by RT-PCR in wild type (WT), *31k*, *48k*, and *65k* mutant plants. Exons and introns are represented as gray boxes and thin lines, respectively, and the positions of primers used are indicated by thick lines. The sequences of primer sets are shown. Identical results were obtained from three independent experiments, one of which is shown.

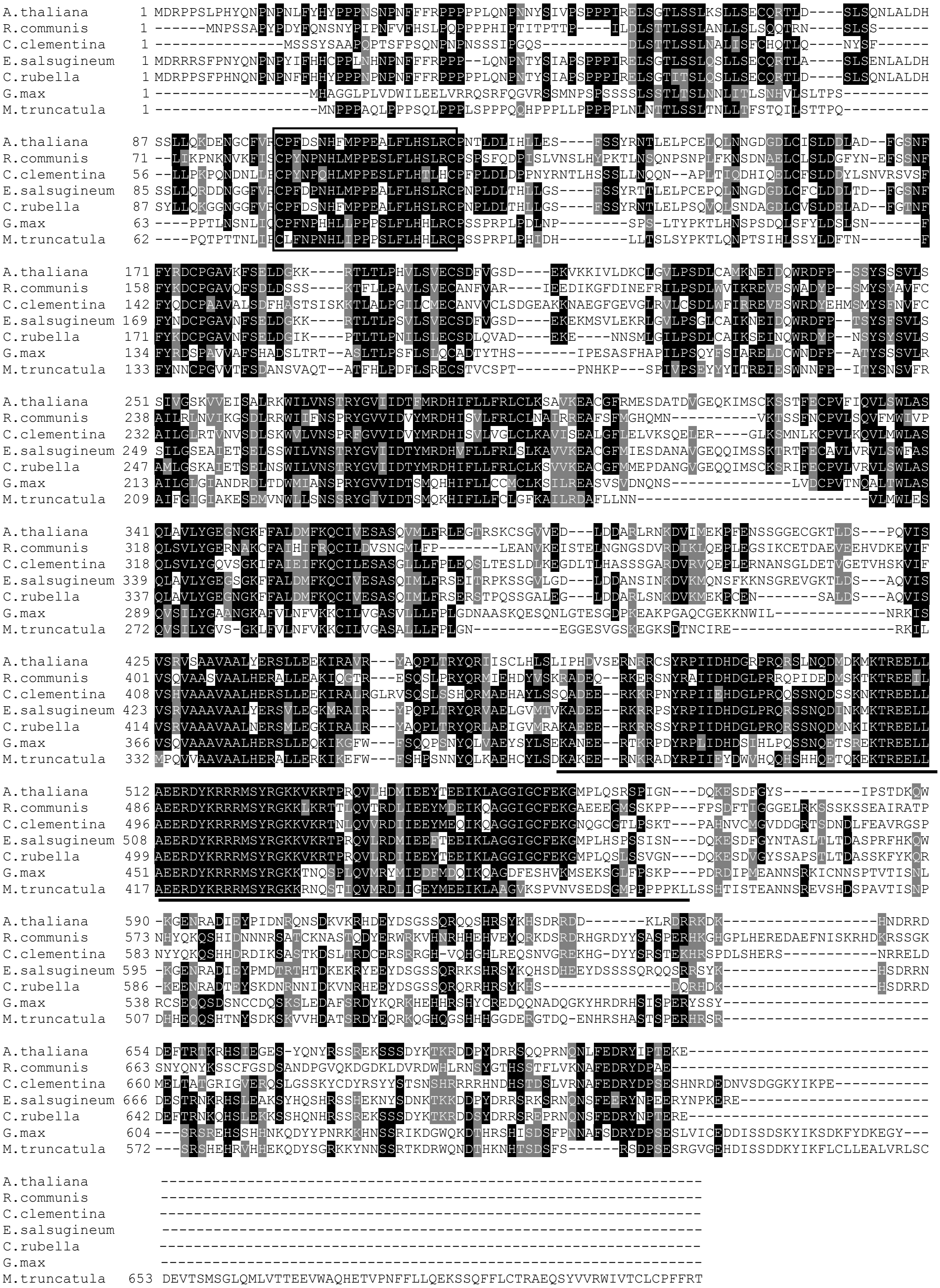

Supplementary Fig. S1

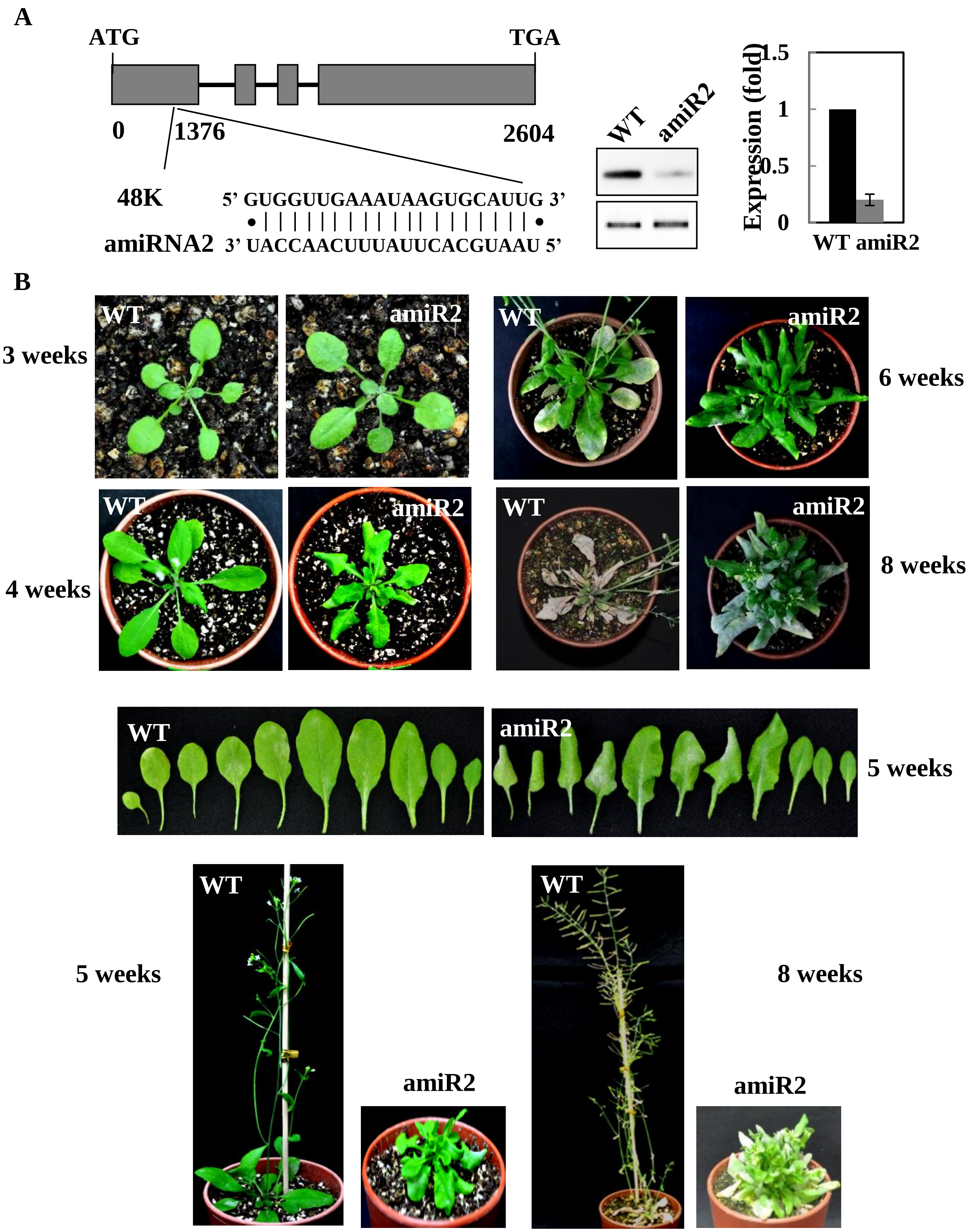

Supplementary Fig. S2

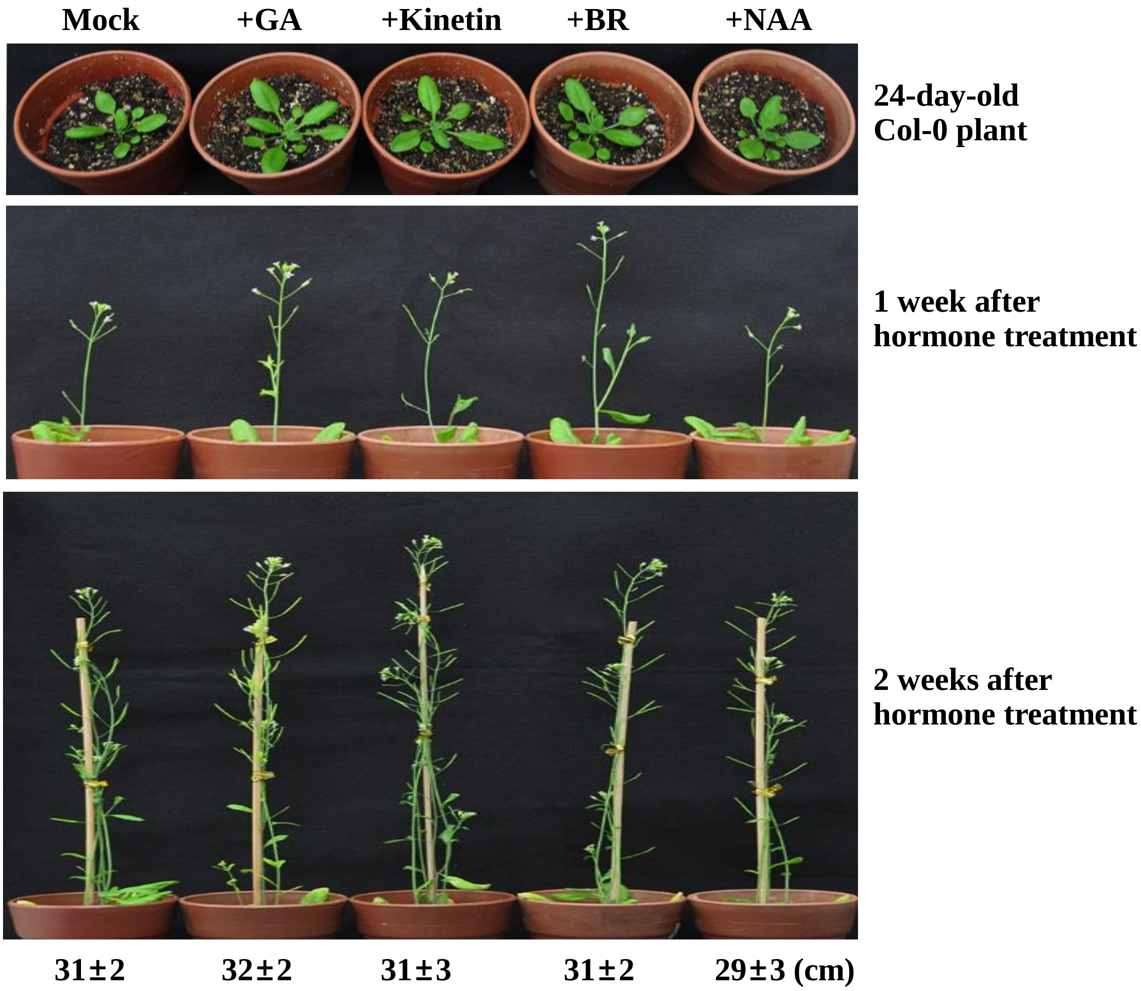

Supplementary Fig. S3

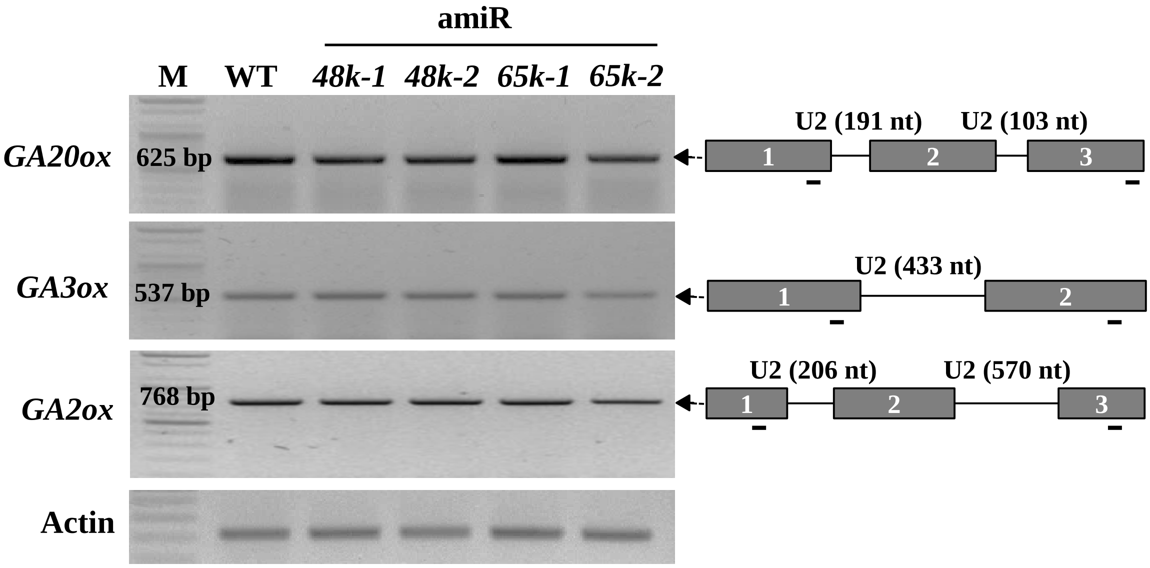

GA20ox Forward: GATTACTTCTGCGATGCGTTGGGACATGGG
Reverse: AGATGGGTTTGGTGAGCCAATCTGAAAAGG

GA3ox Forward: CCAACATCACCTCAACTACTG
Reverse: CTCTTTCCATGTCACCGATTG

GA2ox Forward: GCAGGAGGCTATTGGCTTCTTC
Reverse: CCAAGTCTATAATCACCAAGC

Actin Forward: CAGCAGAGCGGGAAATTGTAAGAG
Reverse: TTCCTTTCAGGTGGTGCAACGAC

Supplementary Fig. S4

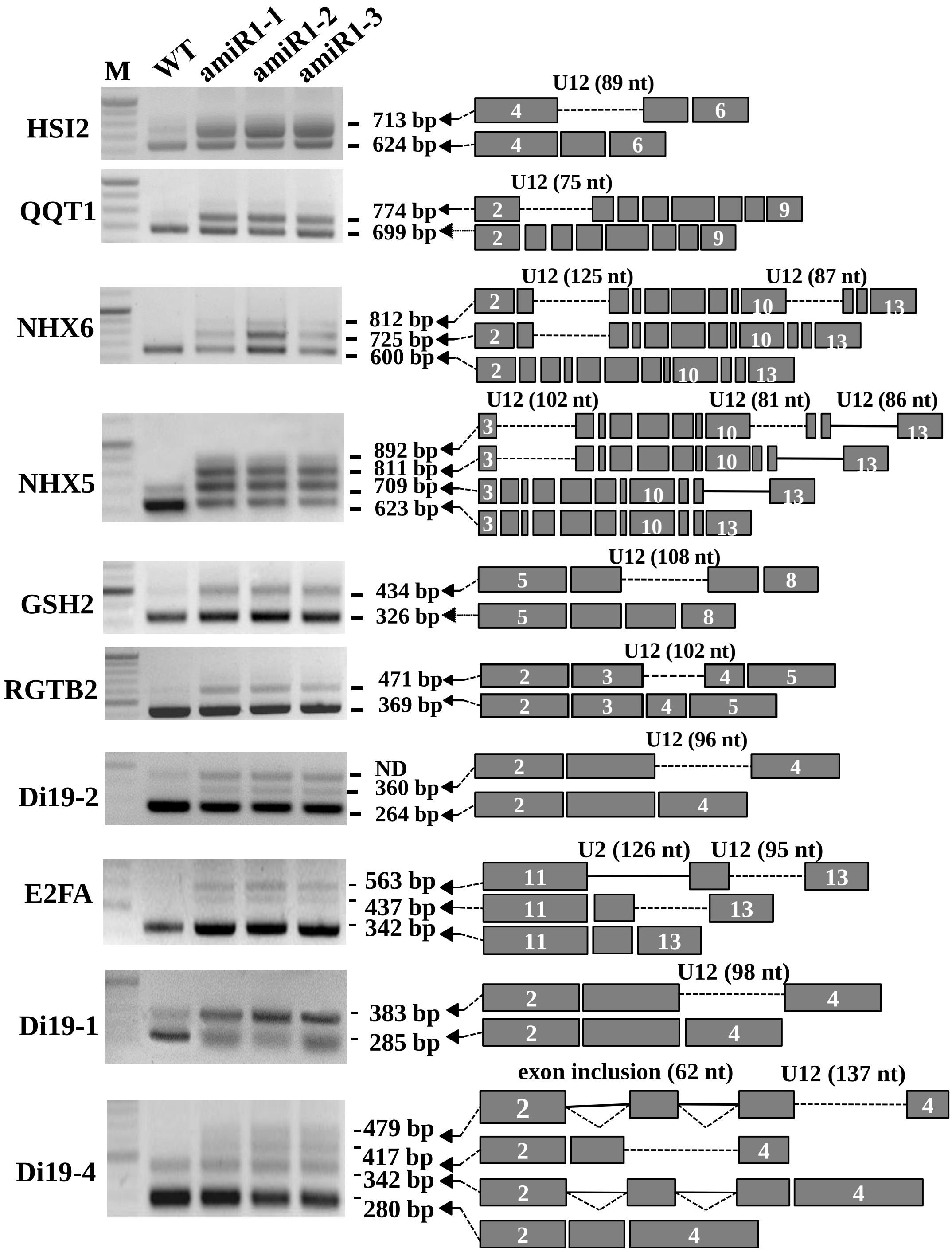

Supplementary Fig. S5

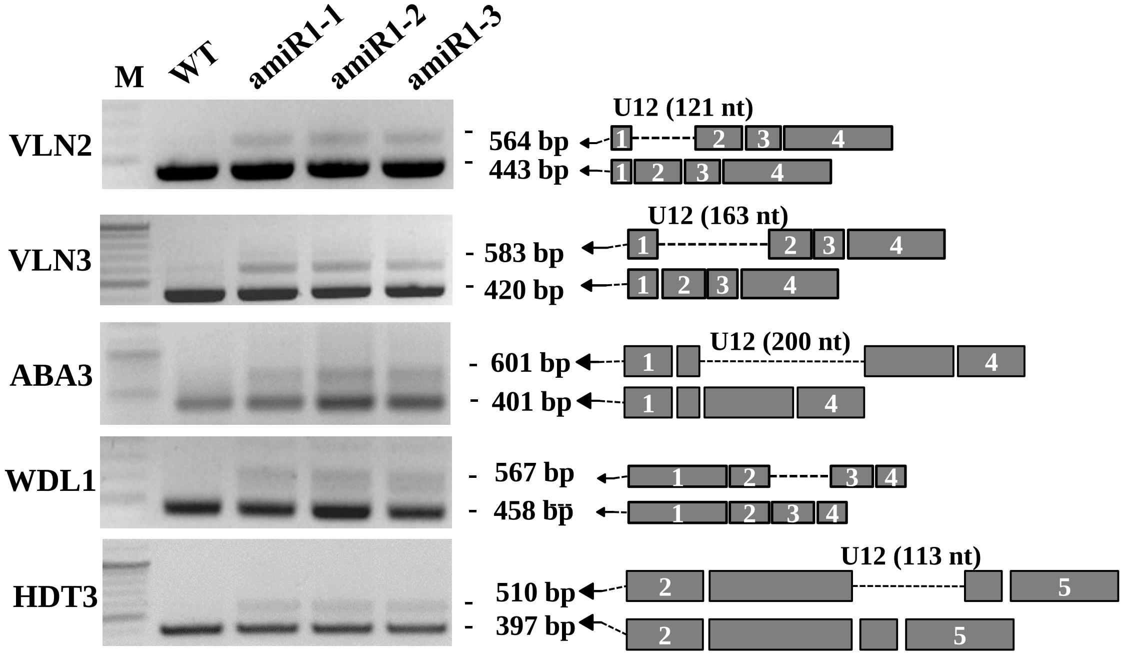

Supplementary Fig. S5 (Continued)

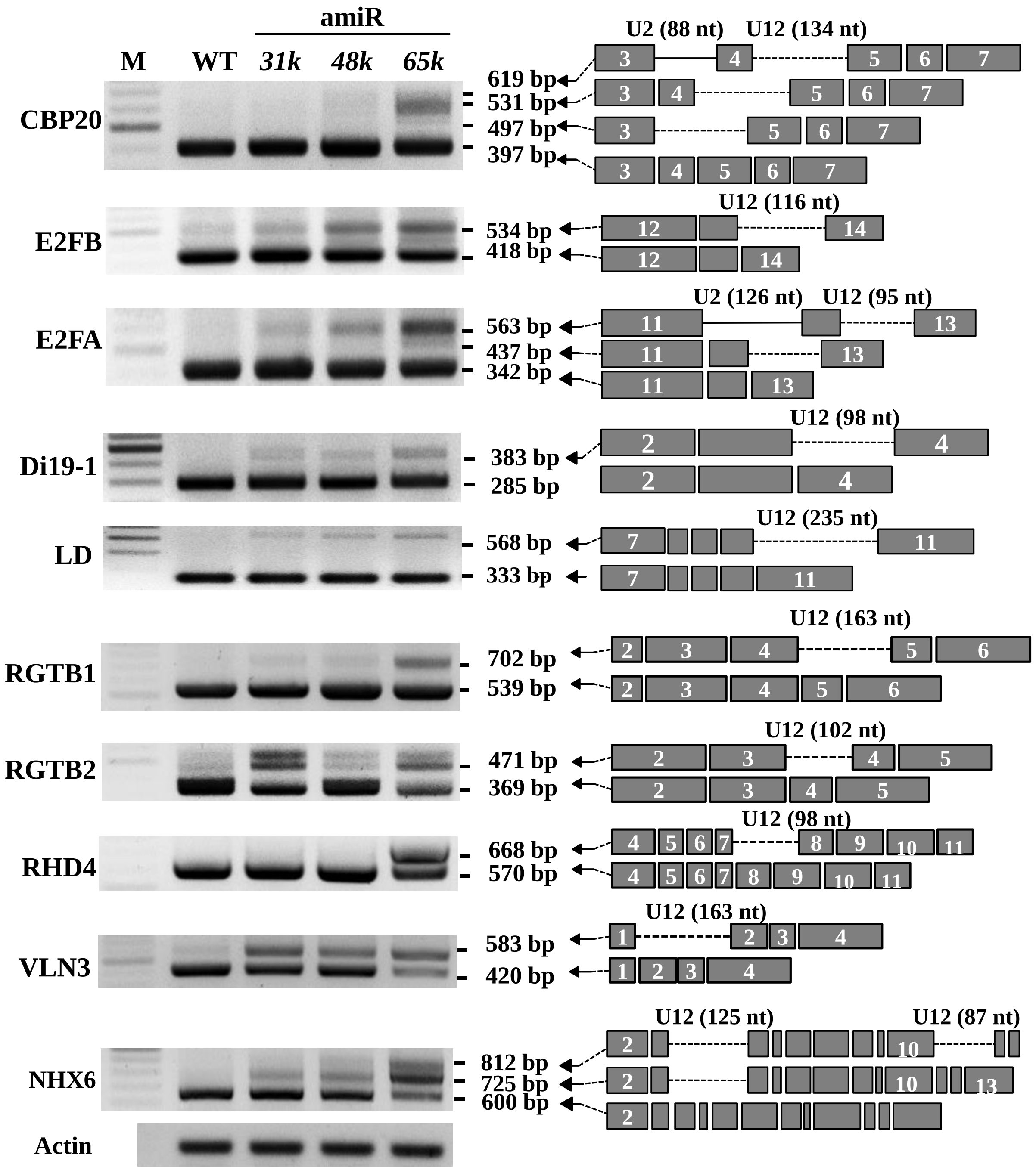

Supplementary Fig. S6

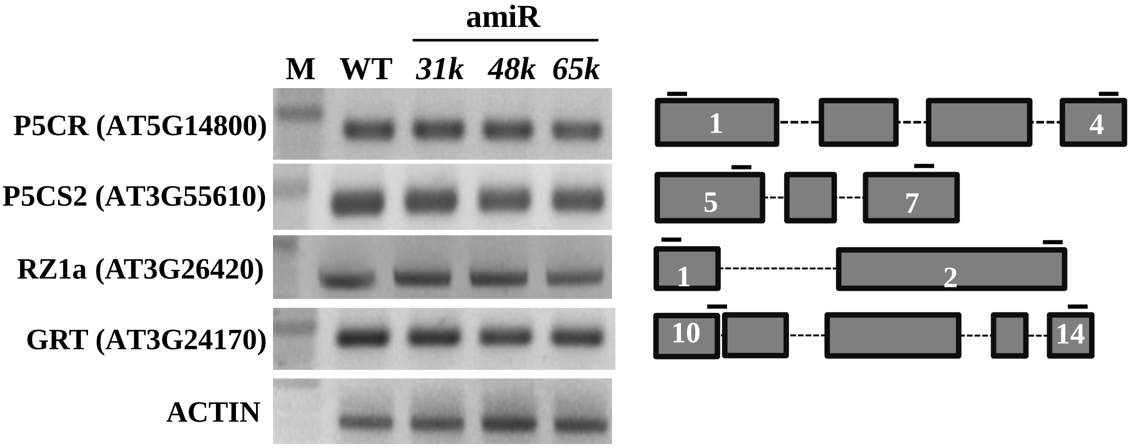

P5CR Forward: 5' TCCGGCGGAGAGTTTCAAGGTAG 3'
P5CR Reverse: 5' CAGCGGCAGGTGTATTAGGCATC 3'

P5CS2 Forward: 5' TGATGCTATAAGCACTCGCAGAGCC 3'
P5CS2 Reverse: 5' TTGATGGAACAGGGTACCAACACG 3'

RZ1a Forward: 5' ATGTCTGAAGATCCGGAG 3'
RZ1a Reverse: 5' GTGGAAACCACCAGCTCTG 3'

GRT Forward: 5' AGGCTGGAGCTGTGAAGGTTGAC 3'
GRT Reverse: 5' CATGATCTCAGCTGCATCAGGACC 3'

Supplementary Fig. S7
